# Supplementary material for: Sulfide:quinone oxidoreductase drives mitochondrial supersulfide metabolism to regulate bioenergetics and longevity in eukaryotes
Source: bioRxiv. 2026 Apr 7:2026.04.05.716515. Preprint. [Version 1] doi: 10.64898/2026.04.05.716515 (PMC13082054; doi:10.64898/2026.04.05.716515)
Supplement: 1 [file NIHPP2026.04.05.716515V1-supplement-1.pdf]

bioRxiv preprint doi: https://doi.org/10.64898/2026.04.05.716515; this version posted April 7, 2026. The copyright holder for this preprint (which was not certified by peer review) is the author/funder, who has granted bioRxiv a license to display the preprint in perpetuity. It is made available under aCC-BY 4.0 International license.

Supplementary Information

Sulfide:quinone oxidoreductase drives mitochondrial supersulfide metabolism to regulate bioenergetics and longevity in eukaryotes

Jia Yao[a,1], Tetsuro Matsunaga[a,b,1], Akira Nishimura[c,1], Meg Shieh[d,1], Tomoaki Ida[a], Minkyung Jung[a], Seiryo Ogata[e,f], Tsuyoshi Takata[a], Uladzimir Barayeu[a,f], Hozumi Motohashi[g], Masanobu Morita[e,*], Takaaki Akaike[a*]

Corresponding authors:

Masanobu Morita, Department of Environmental Medicine and Molecular Toxicology, Tohoku University Graduate School of Medicine, 2-1 Seiryo-machi, Aoba-ku, Sendai 980-8575, Japan. Tel: +81-22-717-8105; Fax: +81-22-717-8219; E-mail: masanobu.morita.e1@tohoku.ac.jp

Takaaki Akaike, Department of Redox Molecular Medicine, Tohoku University Graduate School of Medicine, 2-1 Seiryo-machi, Aoba-ku, Sendai 980-8575, Japan. Tel: +81-22-717-8165; Fax: +81-22-717-8219; E-mail: takaaki.akaike.b4@tohoku.ac.jp

This PDF file includes:

Supplementary Figures 1 to 4

bioRxiv preprint doi: https://doi.org/10.64898/2026.04.05.716515; this version posted April 7, 2026. The copyright holder for this preprint (which was not certified by peer review) is the author/funder, who has granted bioRxiv a license to display the preprint in perpetuity. It is made available under aCC-BY 4.0 International license.

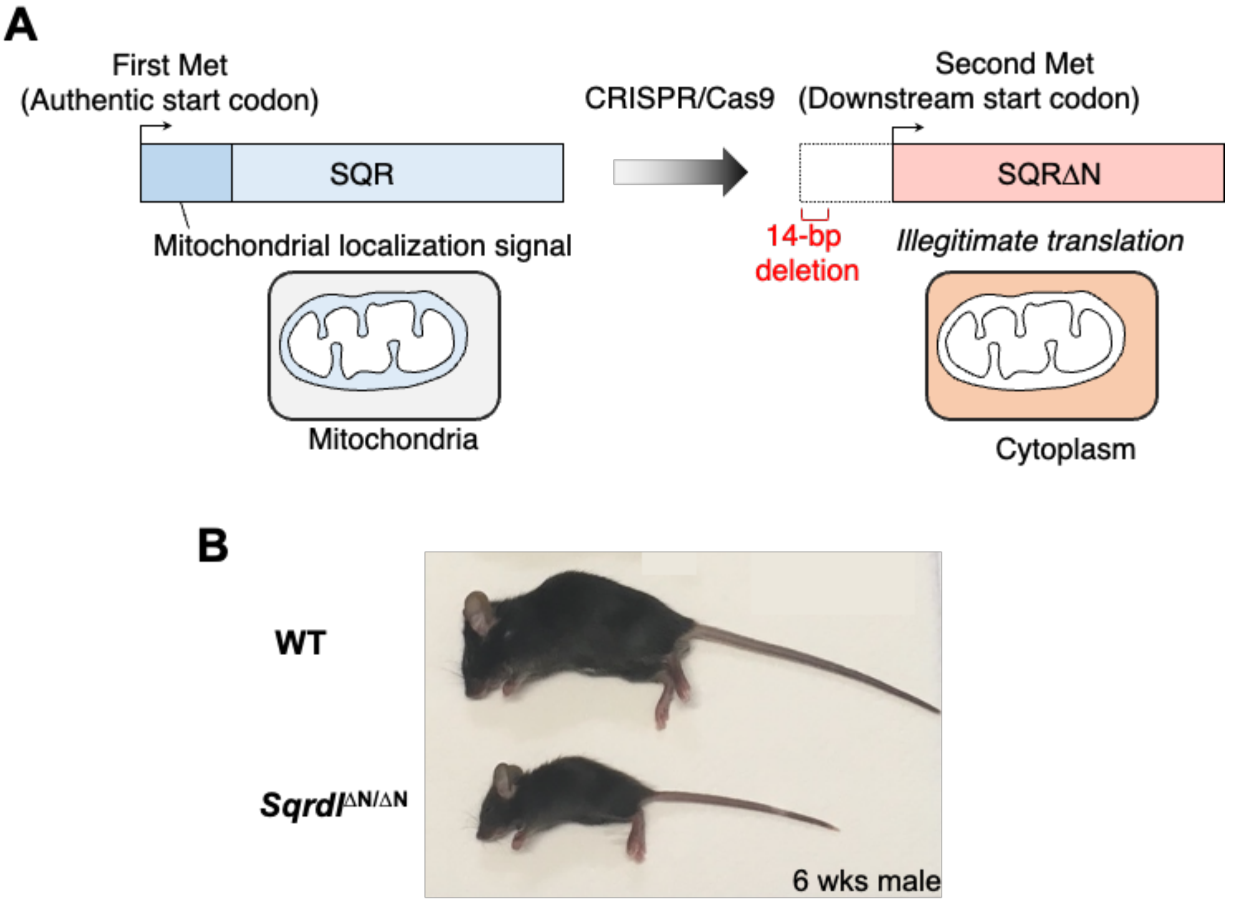

Supplementary Fig. 1. Generation of SQR mutant mice. (A) Schematic presentation of SQR proteins expressed by WT and SQRΔN alleles. (B) Macroscopic appearance of 6-week-old WT, and *Sqrdl*$^{\Delta N/\Delta N}$ littermate male mice.

bioRxiv preprint doi: https://doi.org/10.64898/2026.04.05.716515; this version posted April 7, 2026. The copyright holder for this preprint (which was not certified by peer review) is the author/funder, who has granted bioRxiv a license to display the preprint in perpetuity. It is made available under aCC-BY 4.0 International license.

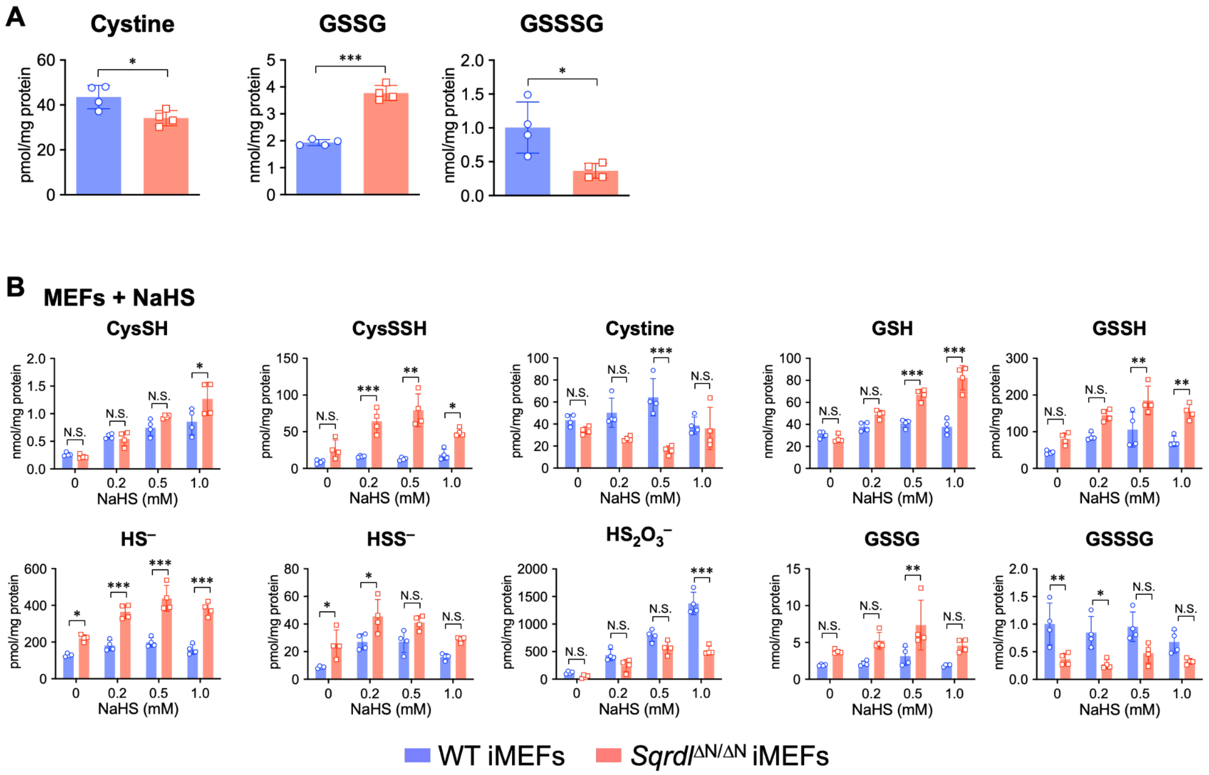

Supplementary Fig. 2. Sulfur metabolome analysis of WT and *Sqrdl*$^{\Delta N/\Delta N}$ iMEFs. (A,B) Quantification of sulfur metabolites in (A) iMEFs, (B) iMEFs treated with NaHS or without such treatment. Data are represented as mean ± s.d. (*n* = 3-5). **P* < 0.05, ***P* < 0.01, and ****P* < 0.001, determined by Student's *t*-test.

.

bioRxiv preprint doi: https://doi.org/10.64898/2026.04.05.716515; this version posted April 7, 2026. The copyright holder for this preprint (which was not certified by peer review) is the author/funder, who has granted bioRxiv a license to display the preprint in perpetuity. It is made available under aCC-BY 4.0 International license.

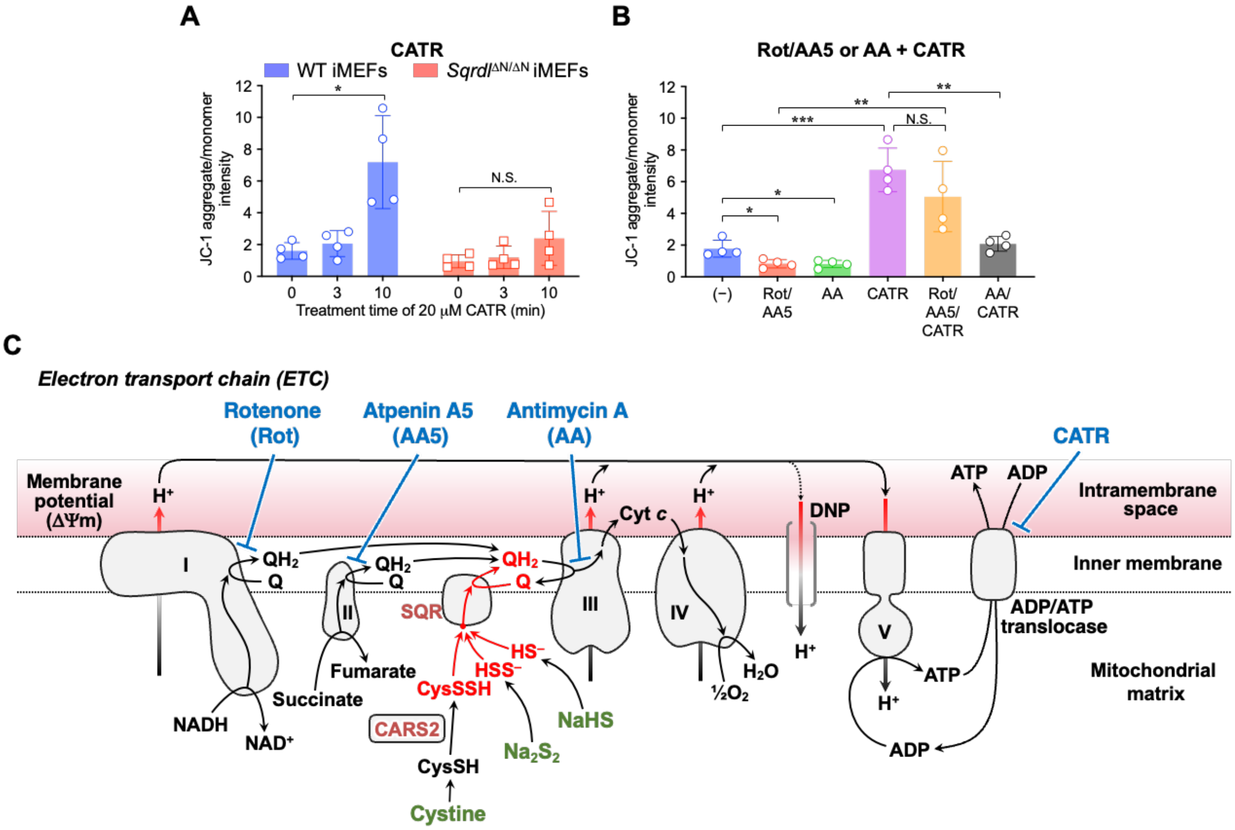

Supplementary Fig. 3. (A) Response of mitochondrial membrane potential to CATR in WT and *Sqrdl*$^{\Delta N/\Delta N}$ iMEFs. (B) Effects of the addition of rotenone (Rot), atpenin A5 (AA5), antimycin A (AA), and CATR on mitochondrial membrane potential of WT iMEFs. Data are mean ± s.d. (n = 3 or 4). **P* < 0.05, ***P* < 0.01, ****P* < 0.001, N.S. (not significant), determined by one-way ANOVA with Tukey's test. (C) Schematic drawing of the ETC in mitochondria with SQR and CARS2. Inhibitors and substrates used in experiments indicated in Fig. 7 appear in blue and green, respectively. Cyt *c*, cytochrome c; ΔΨm, membrane potential.

bioRxiv preprint doi: https://doi.org/10.64898/2026.04.05.716515; this version posted April 7, 2026. The copyright holder for this preprint (which was not certified by peer review) is the author/funder, who has granted bioRxiv a license to display the preprint in perpetuity. It is made available under aCC-BY 4.0 International license.

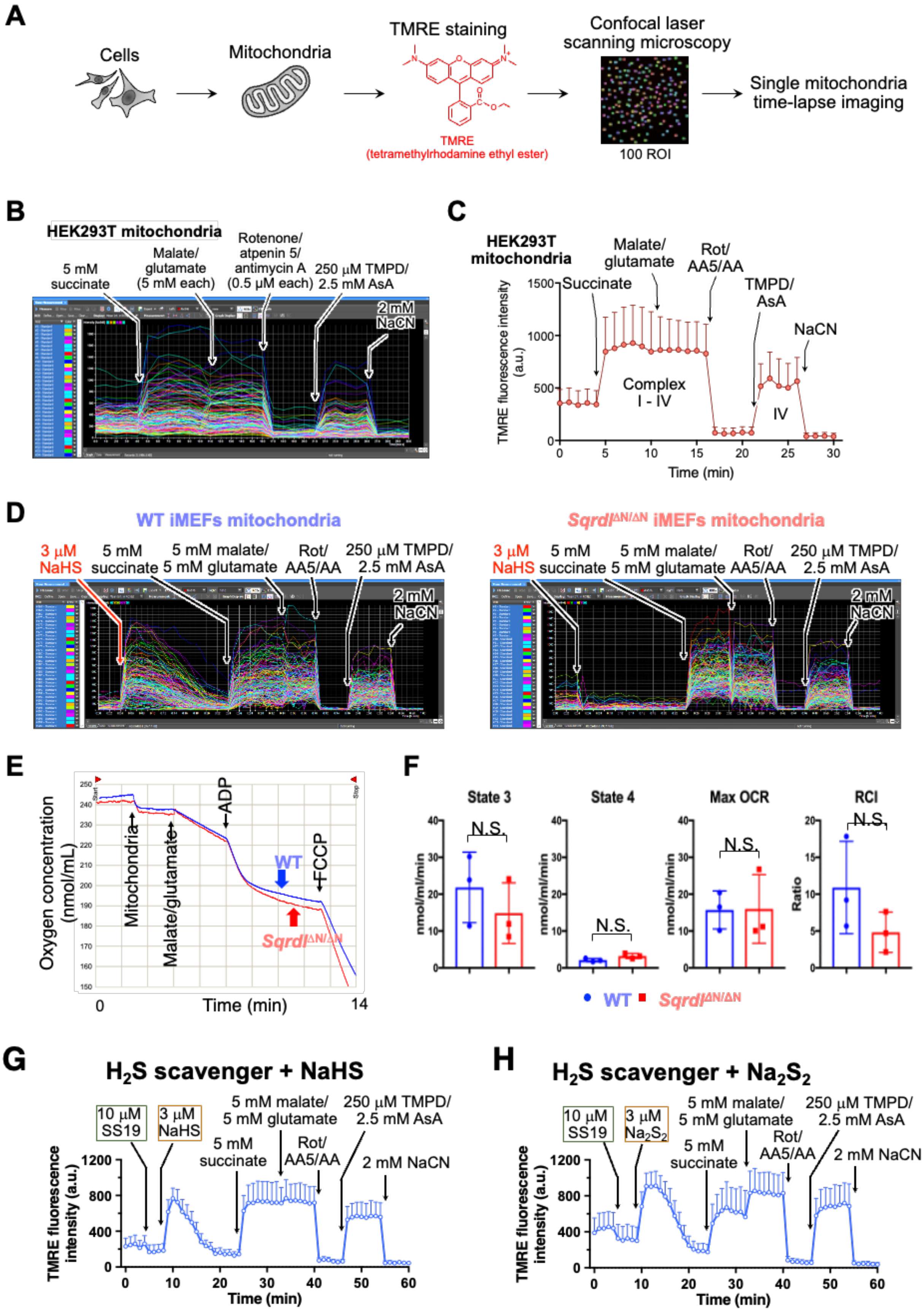

807

bioRxiv preprint doi: https://doi.org/10.64898/2026.04.05.716515; this version posted April 7, 2026. The copyright holder for this preprint (which was not certified by peer review) is the author/funder, who has granted bioRxiv a license to display the preprint in perpetuity. It is made available under aCC-BY 4.0 International license.

Supplementary Fig. 4. Evaluation of the SQR contribution to mitochondrial activity in isolated mitochondria. (A-C) Scheme and representative images of time-lapse changes in TMRE fluorescence of mitochondria isolated from HEK293T cells. (D) Representative images of time-lapse changes in TMRE fluorescence of mitochondria isolated from WT and *Sqrdl*$^{\Delta N/\Delta N}$ iMEFs. (E) Representative oxygen consumption curves of mitochondria isolated from the livers of WT and *Sqrdl*$^{\Delta N/\Delta N}$ mice. (F) OCR and RCI of WT and *Sqrdl*$^{\Delta N/\Delta N}$ mitochondria. OCR at states 3 and 4 and the maximum OCR with uncoupler FCCP from three independent experiments. The RCI was obtained from the ratio of state 3 OCR to state 4 OCR. (G, H) NaHS-induced (G) and Na_2S_2-induced (H) membrane potential in mitochondria isolated from WT iMEFs in the presence of 10 μM SS19, a hydrogen sulfide scavenger. Data are represented as mean ± s.d.
